# Evaluation of a real-time hospital surveillance system for respiratory syncytial virus, Ontario, Canada, 2022–2023

**DOI:** 10.14745/ccdr.v51i08a02

**Published:** 2025-08-28

**Authors:** Michelle Murti, Ania Sarnocinska, Mahnaz Alavinejad, Aidin Kerem, Kamil Malikov, Kevin Brown, Tiffany Fitzpatrick, Michael Hillmer

**Affiliations:** 1 Ministry of Health, Toronto, ON; 2 Dalla Lana School of Public Health, University of Toronto, Toronto, ON; 3 Public Health Ontario, Toronto, ON; 4 Institute for Health Policy, Management and Evaluation, University of Toronto, Toronto, ON

**Keywords:** respiratory syncytial virus, hospitalizations, surveillance, evaluation

## Abstract

**Background:**

Respiratory syncytial virus (RSV) surged in the 2022–2023 respiratory season after low activity during the pandemic. To monitor the RSV season in real time and support healthcare planning, Ontario introduced daily hospital bed census reporting of RSV hospitalizations by age group (0–17, 18–64, 65 years and older).

**Objectives:**

To assess the completeness and quality of the newly introduced real-time surveillance compared to end-of-season ICD-10 coded hospitalization discharge abstract data (DAD) from November 22, 2022, to March 31, 2023.

**Methods:**

Respiratory syncytial virus hospitalizations from both data sources were compared to RSV laboratory positivity to assess concordance with overall RSV activity. A longitudinal comparison by age group was assessed by time-lagged cross-correlation of the daily submission data versus DAD data, including cross correlation coefficients for each time lag, confidence bound and the highest correlation value.

**Results:**

Both data sources followed trends in RSV positivity. Data by age groups showed an early peak of paediatric admissions followed by a peak in adult and older adult hospitalizations. Daily surveillance consistently underestimated hospitalizations with a peak of 430 beds by DAD on January 7, 2023, versus 322 beds (75%) for daily reporting on the same day. The maximum correlation coefficient values were 0.67 (all ages), 0.57 (0–17 years), 0.66 (18–64 years) and 0.63 (65 years and older).

**Conclusion:**

Implementation of daily hospital reporting provided accurate trending in RSV hospitalizations by age group to inform within season healthcare and public health planning.

## Introduction

Respiratory syncytial virus (RSV) is one of the most common causes of respiratory infection among children under five years old globally, with over 100,000 RSV-attributable deaths worldwide annually ((1)). Older adults are also susceptible to morbidity and mortality from RSV, with an estimated 470,000 hospitalizations in adults 60 years and older in high-income countries ((2)). In Ontario, RSV hospitalization rates for children younger than five years old is 4.2 per 1,000 person-years, and 29.6 per 1,000 person-years for infants aged one month old ((3)). During the COVID-19 pandemic, non-pharmaceutical interventions interrupted usual seasonal patterns of respiratory infections, and there had been very low levels of RSV during the 2020–2021 respiratory season in Ontario ((4,5)). However, by mid-2022, there were indications of a significant RSV resurgence globally, and an early and severe season in Australia, with high morbidity among children younger than five years old ((6)). Along with early and elevated cases of influenza and the ongoing COVID-19 pandemic, the “triple threat” was causing significant strain on the healthcare system in Australia and other parts of the southern hemisphere ((7)). Given this pattern of illness, there was concern for a similar early respiratory season in the fall/winter of 2022–2023 in the northern hemisphere with disproportionate impacts on the paediatric population from overlapping peaks of RSV, influenza and COVID-19 that could overwhelm the paediatric healthcare system.

Individual cases of RSV are not reportable to public health authorities in Ontario, Canada. Surveillance is based on reporting of RSV outbreaks in institutions and public hospitals to public health authorities ((8)), and testing of data from hospital and public health laboratories based on respiratory multiplex testing of admitted patients as well as paediatric patients in the emergency department ((9)). Real-time surveillance for severity impacts from RSV did not exist in Ontario, resulting in an inability to detect, within the season, healthcare system surges related to RSV. Therefore, in the fall of 2022, in anticipation of atypically early and high levels of RSV, the Ontario Ministry of Health added daily hospital bed census reporting of admissions and cases for RSV by age group to the ongoing COVID-19 and influenza daily bed census reporting by hospitals. Initiation of real-time surveillance subsequently enabled the Ministry of Health to develop forecasting models for public health and health system decision-making throughout the 2022–2023 season. While there was high utility from the newly implemented surveillance system for informing public health and healthcare decision-making, the completeness and quality of the newly implemented data reporting were unknown.

The primary objective of this study was to evaluate the completeness and cross-correlation of the newly implemented hospital-reported daily bed census reporting data on RSV admissions by age group in Ontario to validated RSV-coded hospitalization discharge data.

## Methods

### Data sources

Hospital-reported bed census data was obtained from the Ontario Ministry of Health, including the daily number of hospital beds occupied by RSV patients by age group (0–17, 18–64, 65 years and older) from initiation of the surveillance on November 24, 2022, until March 31, 2023, when RSV activity returned to inter-seasonal levels. All 138 acute care hospital sites in Ontario were instructed to submit daily the total number of beds occupied by RSV inpatients each day using the following question: “As of 12 midnight, what is the total number of confirmed RSV inpatients in your facility?” There were approximately 20,000 total acute care beds amongst the 138 sites. Data submitted were based on data accurate as of the date they were reported and were not subsequently updated or corrected.

Data from the Canadian Institute for Health Information’s Discharge Abstract Database (CIHI DAD) was obtained for all acute care separations in Ontario from November 24, 2022, to March 31, 2023, with ICD-10 codes J12.1, J21.0, J20.5 or B97.4 in any of the diagnostic fields. The CIHI DAD data were linked to the Ontario Registered Persons Database file to assign age group categories for admissions (0–17, 18–64, 65 years and older). Admissions that occurred after midnight and discharged prior to the following midnight (i.e., length of stays fewer than 24 hours) were not included in the hospital-reported daily data; therefore, corresponding CIHI DAD RSV admissions were obtained by removing admissions of fewer than two calendar days.

Respiratory syncytial virus testing data by age group (0–17, 18–64, 65 years and older) for the period of September 1, 2022, to March 31, 2023, was obtained from the Provincial Public Health Laboratory System. Individuals with unknown age were excluded from the dataset. All specimens and test methods for RSV were included for calculation of daily percent positivity by age group. Admissions were compared to laboratory testing positivity over the reporting period for descriptive analysis of hospitalizations relative to RSV activity.

### Analysis

Respiratory syncytial virus rates based on the daily bed census data by age group were calculated per 100,000 population using Ontario population estimates for the year 2022 projected from the 2016 census. Peak bed volume days by age group were compared between the two datasets to assess completeness.

A longitudinal comparison of the data sources for all of Ontario and by age group was assessed by time-lagged cross-correlation of the daily submission data versus CIHI DAD data. The augmented Dickey-Fuller (ADF) test showed that the time series was not stationary. The 28-day rolling average was subtracted to detrend both series for an accurate cross-correlation analysis. The cross-correlation coefficients for each time lag, including the confidence bound and the highest correlation value, was assessed with coefficients >0.5–1.0 indicating strong correlation.

All analysis was completed using SAS EG Version 7.13 and Python 3.10.

### Data access and ethics approval

Public Health Ontario’s Research Ethics Board assessed this study to be of minimal risk and waived the requirement for review.

## Results

**Figure 1:**
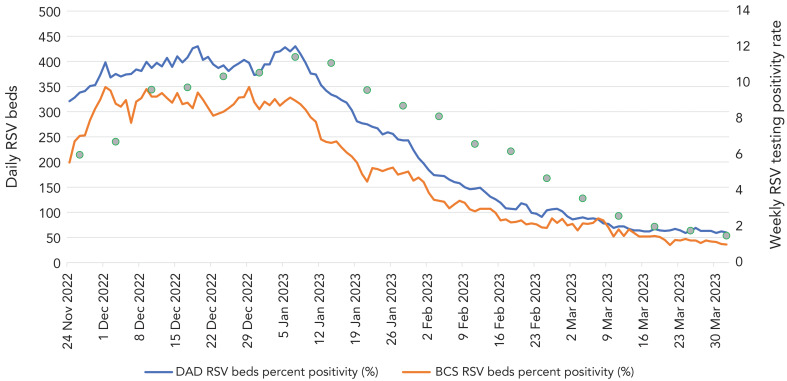
Daily number of hospital beds occupied by patients with respiratory syncytial virus (RSV) hospitalizations, as compared to the provincial daily RSV testing positivity rate, November 22, 2022–March 31, 2023^a^ Abbreviations: BCS, bed census surveillance; DAD, Discharge Abstract Database; RSV, respiratory syncytial virus ^a^ As reported by daily real-time reporting during the season, and by Canadian Institute for Health Information Discharge Abstract Database

**Figure 1** shows daily RSV hospitalizations of all ages in Ontario based on the daily hospital-reported data and the CIHI DAD data over the 2022–2023 respiratory season, along with RSV activity in the province based on provincial RSV positivity. As hospital RSV reporting was initiated later in the season, hospital admissions were already high at the end of November and continued to remain elevated until early January and began declining steadily to inter-seasonal levels by the end of February 2023. This corresponded to RSV positivity in the province that peaked in the first week of January 2023 and then declined steadily. While hospital-reported RSV bed counts followed the same trend fluctuations as CIHI DAD data, they were consistently lower than CIHI DAD admissions throughout the season, with a peak of 430 CIHI DAD beds on January 7, 2023, versus 322 (75%) on the same day from hospital reporting. While hospitals were instructed to start reporting as of November 24, 2022, hospital-reported beds appear to rise rapidly over the first week of reporting as more hospitals began reporting.

**Figure 2:**
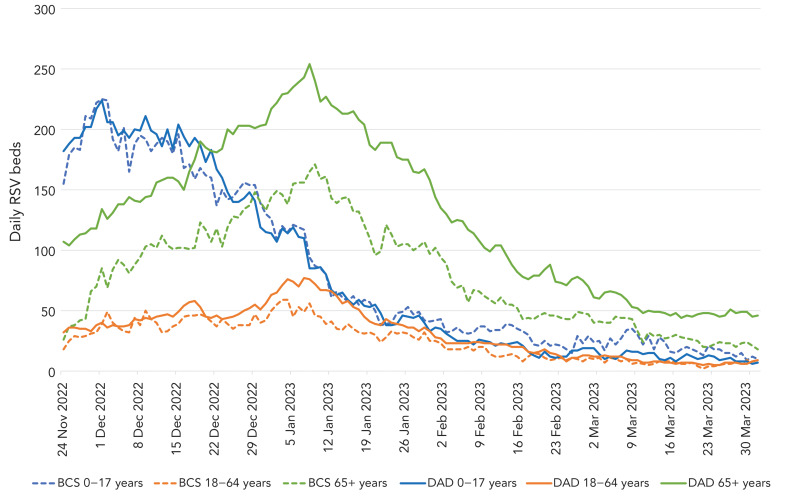
Daily number of hospital beds occupied by patients with respiratory syncytial virus by age group^a^ hospitalizations, November 22, 2022–March 31, 2023^b^ Abbreviations: BCS, bed census surveillance; DAD, Discharge Abstract Database; RSV, respiratory syncytial virus ^a^ Age groups were 0–17, 18–64 and 65 years and older ^b^ As reported by daily real-time reporting during the season, and by Canadian Institute for Health Information Discharge Abstract Database

**Figure 2** shows RSV admissions in both data systems by reported age groups. When reporting started at the end of November 2022, RSV admissions were driven by those 0–17 years of age, with a peak at the beginning of December 2022 (peak 224, CIHI DAD), followed by a steady decline for the rest of the season. After the end of January, daily bed reporting for paediatric beds was higher than CIHI DAD data, although total beds from both data sources were low. Respiratory syncytial virus admissions among individuals aged 65 years and older rose steadily from the start of reporting until early January 2023 (peak 254, CIHI DAD), declining through the rest of the season. Admissions for adults 18–64 years of age peaked at a similar time as admissions among those aged 65 years and older and contributed a small proportion of total RSV admissions (peak 77, CIHI DAD) throughout the season. Daily reported admissions for those aged 65 years and older were consistently undercounted compared to CIHI DAD data throughout the season, with a peak bed count of 254 by CIHI DAD on January 8, 2023, versus 165 beds (65%) reported by hospitals that day.

**Figure 3:**
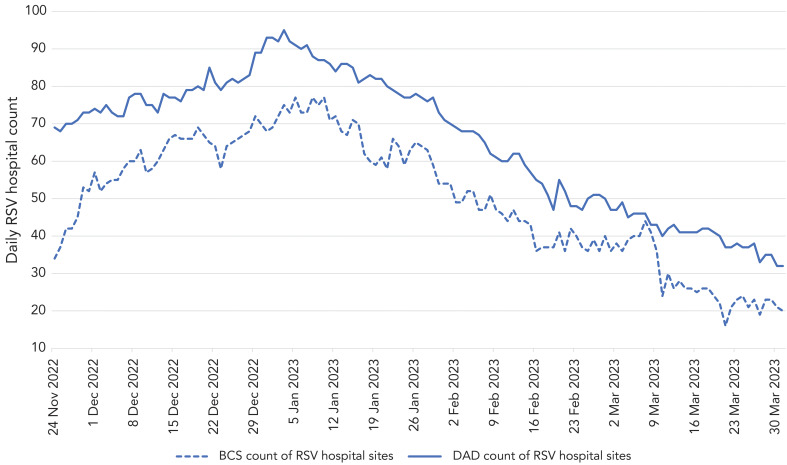
Number of hospital sites represented in daily respiratory syncytial virus admissions, all ages, November 22, 2022–March 31, 2023^a^ Abbreviations: BCS, bed census surveillance; DAD, Discharge Abstract Database; RSV, respiratory syncytial virus ^a^ As reported by daily real-time reporting during the season, and by Canadian Institute for Health Information Discharge Abstract Database

To assess the impact of non-reporting by some hospitals, **Figure 3** shows the number of hospital sites reporting in the daily real-time surveillance, as compared to the number represented each day in the CIHI DAD data. There was an initial ramp-up phase through November to mid-December, when more hospitals started participating in the daily bed reporting. Throughout the season, hospitals contributing to the daily real-time surveillance were consistently lower than hospitals in CIHI DAD data. There was also a decline in the hospitals providing daily surveillance data at the end of December, corresponding to the peak holiday period in Ontario.

**Figure 4:**
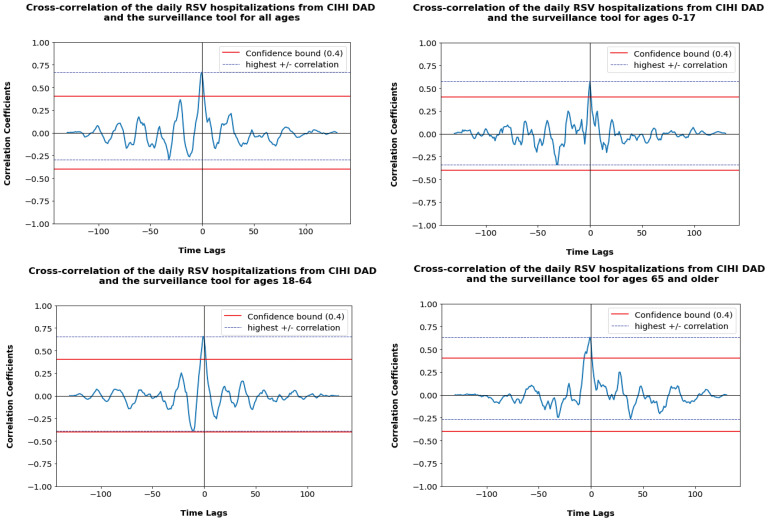
Cross-correlation plots after detrending data on respiratory syncytial virus hospitalizations for all age groups^a,b^ Abbreviations: BCS, bed census surveillance; CIHI DAD, Canadian Institute for Health Information Discharge Abstract Database; RSV, respiratory syncytial virus ^a^ Detrending for the daily real-time surveillance compared to Canadian Institute for Health Information Discharge Abstract Database data ^b^ Age groups were 0–17, 18–64 and 65 years and older

Cross-correlation analysis found that the highest value of the correlation coefficient is obtained at lag=0 for all ages and for ages 0–17 years, and at lag=−1 for ages 18–64 years and 65 years and older. The maximum correlation coefficient values were 0.67 (all ages), 0.57 (0–17 years), 0.66 (18–64 years) and 0.63 (65 years and older). **Figure 4** shows the cross-correlation plots after detrending by age group.

## Discussion

Respiratory syncytial virus contributed to substantial pressures on the paediatric healthcare system in the 2022–2023 respiratory season, along with influenza and COVID-19 ((10)). Initiation of Ontario hospital daily bed census reporting in November 2022 for RSV admissions enabled real-time surveillance of its impacts on the healthcare system over the season. The Ministry of Health and health system partners were able to utilize this surveillance to inform hospital mitigation measures, particularly in paediatric hospitals that experienced significant pressures between November–December 2022 ((11)).

This evaluation demonstrates that the newly implemented real-time surveillance provided strong correlation (coefficients >0.5–1) with overall trends in RSV admissions among the different age groups when compared to ICD-10-coded admission data ((12)). Real-time hospital-based surveillance underrepresented the true magnitude of the hospital pressures, as it was consistently lower than CIHI DAD admissions for adults and older adults throughout the reporting period, and represented only 75% of beds at the peak day for CIHI DAD beds. Conversely, daily census reporting overestimated hospital beds for paediatric patients in the later part of the season from late January 2023 to the end of the reporting period, when total beds by both data sources were low (fewer than 50 beds daily).

Overall, under-representation was mostly driven by beds for those 65 years and older, where, on the peak day according to CIHI DAD data, hospital-reported beds were only 65%. The undercounting was at least partly due to incomplete submissions, as there were consistently fewer hospitals reporting compared to all acute care hospitals with RSV-admitted patients in the CIHI DAD data. Hospital reporting may also be lower than CIHI DAD data due to delays in RSV laboratory result reporting, where patients were admitted but had not yet been identified as an RSV-related admission. Additionally, hospitals may have omitted admissions where RSV was not the most responsible diagnosis and only a contributing diagnosis in their daily reporting. Potential reasons for overestimation of paediatric beds are less clear, but may reflect syndromic clinical diagnoses of RSV in admitted patients that were not coded as RSV admissions in CIHI DAD.

As RSV hospitalizations are reportable neither nationally nor in Ontario, there are no surveillance systems for RSV admissions to guide within season healthcare and hospital planning. In the United States, RSV hospitalization surveillance is conducted by RSV-NET, a network of sites across 12 states ((5)). Sentinel-based surveillance, such as RSV-NET, provides important real-time information on epidemiological trends, but is insufficient to support fulsome hospital capacity planning within the season as reporting is only representative and does not capture all hospitals. As far as we are aware, this analysis is the first report assessing the implementation of a province-wide real-time RSV hospitalization surveillance system.

### Limitations

Limitations of the analysis include the incompleteness of the data in that daily reporting surveillance only began at the end of November 2022, when RSV activity was already high. While it is possible to assess which hospitals reported RSV hospitalizations in real time, it is not possible to fully distinguish whether hospitals did not report because they omitted reporting, or if there were “zero” admissions reported that day. As aggregate data were not provided by sex or narrower age bands, more refined analyses of sex differences and impacts in children younger than one year or younger than five years of age were not available for analysis.

While this analysis has shown strong correlation of trends and reasonable completeness of the newly implemented real-time daily hospital reporting, it does not include an assessment of other aspects of a surveillance system, such as feasibility and acceptability of reporting. Daily reporting by all hospital sites is time-consuming and human-resource intensive. The provision of the data throughout the season is an additional demand on hospitals; however, hospitals have also recognized the value of the data in providing intelligence locally, regionally and provincially regarding hospital capacity for planning and resource management purposes. At a provincial level, the data have been leveraged to support weekly forecasting of hospital bed projections for RSV, along with COVID-19 and influenza, to support senior-level decision-making at the Ontario Ministry of Health and Ontario Health. Ongoing reporting has also been incorporated into publicly reported provincial surveillance on RSV hospital bed occupancy as part of severe outcome surveillance of respiratory viruses in Ontario ((4)). Future evaluations are needed to assess the feasibility, acceptability, costs and sustainability of this surveillance system, and an assessment of completeness and correlation in the 2023–2024 and 2024–2025 seasons.

## Conclusion

Implementation of a province-wide real-time surveillance system for RSV hospitalizations in Ontario in the fall of 2022 was a successful initiative providing reliable and accurate trending by age groups over the respiratory season. Compared to ICD-10-coded hospital admissions, the real-time surveillance under-reported total admissions, particularly for those aged 65 years and older, that should be taken into consideration for within-season hospital bed planning. Without any other real-time surveillance to provide data on RSV admissions, these data provide valuable insights for Ontario to guide local, regional and provincial level hospital planning during the respiratory season.
